# A consensus-based template for uniform reporting of data from pre-hospital advanced airway management

**DOI:** 10.1186/1757-7241-17-58

**Published:** 2009-11-20

**Authors:** Stephen JM Sollid, David Lockey, Hans Morten Lossius, Pre-hospital advanced airway management expert group

**Affiliations:** 1 Department of Research and Development, Norwegian Air Ambulance Foundation, Drøbak, Norway; 2 Air Ambulance Department, Oslo University Hospital, Oslo, Norway; 3 London HEMS, The Royal London Hospital, London, UK; 4 Department of Surgical Sciences, University of Bergen, Norway

## Abstract

**Background:**

Advanced airway management is a critical intervention that can harm the patient if performed poorly. The available literature on this subject is rich, but it is difficult to interpret due to a huge variability and poor definitions. Several initiatives from large organisations concerned with airway management have recently propagated the need for guidelines and standards in pre-hospital airway management. Following the path of other initiatives to establish templates for uniform data reporting, like the many Utstein-style templates, we initiated and carried out a structured consensus process with international experts to establish a set of core data points to be documented and reported in cases of advanced pre-hospital airway management.

**Methods:**

A four-step modified nominal group technique process was employed.

**Results:**

The inclusion criterion for the template was defined as any patient for whom the insertion of an advanced airway device or ventilation was attempted. The data points were divided into three groups based on their relationship to the intervention, including system-, patient-, and post-intervention variables, and the expert group agreed on a total of 23 core data points. Additionally, the group defined 19 optional variables for which a consensus could not be achieved or the data were considered as valuable but not essential.

**Conclusion:**

We successfully developed an Utstein-style template for documenting and reporting pre-hospital airway management. The core dataset for this template should be included in future studies on pre-hospital airway management to produce comparable data across systems and patient populations and will be implemented in systems that are influenced by the expert panel.

## Background

Advanced airway management is a critical intervention that is carried out regularly on the most severely ill or injured patients in the pre-hospital setting. Evidence for its benefit is scarce and of poor quality [1,2], but it is generally accepted that securing a compromised airway in critically ill patients as early as possible is of the highest priority [3]. It has also been established that, when performed poorly, pre-hospital airway management is hazardous and can worsen the outcome [4-7]. Studies on this subject are difficult to interpret because of the huge variability and poor definition of operator experience, technique, and patient case mix [1]. Most published studies that have influenced practice are from pre-hospital systems in North America, where paramedics and nurses usually manage the airway of patients. In Europe, many Emergency Medical Service (EMS) systems are physician-manned, and some studies suggest that this setup is a significant factor in safe and successful pre-hospital airway management [8,9]. There are certainly several key studies that seriously question the safety of paramedic advanced airway management in US systems [4,5,10,11].

A recent initiative from the Scandinavian Society for Anaesthesiology and Intensive care (SSAI) to define a standard for pre-hospital airway management [12] and the recently published guidelines on pre-hospital anaesthesia from The Association of Anaesthetists of Great Britain and Ireland (AAGBI) [13] suggest that there is a demand for guidelines in pre-hospital airway management. There also seems to be a need for standardisation of training and maintenance of critical skills like advanced airway management in established physician-manned pre-hospital systems [14]. A position paper from the National Association of EMS Physicians (NAEMSP) also called for better training in airway management for pre-hospital personnel and a standardisation of protocols [15]. The same organisation has issued recommended guidelines for the reporting of data from pre-hospital airway management within the US system [16]. Implementation of new guidelines or curricula should be accompanied by a quality assessment of the implementation to answer the question of whether the new guidelines or curriculum changes result in better practice.

Recently, a revised Utstein-style template for the uniform reporting of data following a major trauma was published to simplify the comparison of data from different trauma registries [17]. We believe that a similar template for the uniform reporting of data related to pre-hospital airway management will help us to better compare the data and evaluate the implementation of new guidelines or methods. Such a template would allow pre-hospital organisations with different infrastructures to contribute information to the literature, which could then be easily interpreted. It would also allow for the collaboration of key pre-hospital organisations in different countries and systems to produce good quality uniform data and publications on specific areas of pre-hospital airway practice, particularly relating to patient safety and the reduction of adverse incidents. We therefore think that such a template needs to be based on a consensus process supported by a geographically dispersed group of experts. Such a consensus-based template would also be a natural advancement of similar templates developed by national interest organisations [16]. We believe that such a project has the potential to contribute to all elements of the Theoretical Model of Factors in Patient Outcome published by the International Liaison Committee on Resuscitation, the so-called Utstein formula of survival [18].

It has therefore been our goal to initiate and carry out a structured consensus process with invited international experts to establish a set of core data points to be documented and reported in cases of advanced pre-hospital airway management.

## Methods

The template was developed using a four-step, modified nominal group technique (NGT) [19,20].

### The expert panel

We invited physicians from Europe and North America who have contributed substantially to research, the development of guidelines and/or are considered experts in the field of pre-hospital airway. The panel consisted of clinicians, most of who are, or have been, directly involved in pre-hospital care.

### Data point definitions

The data variables need to be clearly defined to prevent misinterpretation. They should also be simple to register and integrate into existing activity registries. A data variable dictionary should contain information on the "data point number", "data point name", "descriptive field name", "type of data", "data point category/value", "definition of data point", "source of data information", and "coding guidance" [17]. The definitions used in the template are adapted to and, in some cases, based on the Utstein template for the uniform reporting of data following major trauma [17] and the recommended guidelines for reporting on emergency medical dispatch when conducting research in emergency medicine [21].

### Core data variables

As with previous Utstein-style templates [17,21], we differentiated between core and optional data variables. We chose to focus on the core data variables, i.e., those data variables that absolutely must be collected. These variables were divided into three groups based on their relationship to the intervention advanced airway management: "system variables", "patient variables", and "post-intervention variables".

#### System variables

The system variables describe the system in which the advanced airway management is performed, meaning the specific characteristics of the pre-hospital EMS in which the procedure is performed. Large differences exist between EMS systems not only globally, but also in relatively homogenous areas like Scandinavia [22]. The system variables should therefore indicate the key differences and allow for a comparison of the effect of a system structure on outcome.

#### Patient variables

Patient variables should describe the patients' conditions before the intervention, specifically physiological variables or scoring systems that describe co-morbidity, severity of injury or illness, or other factors that may influence patient outcome.

#### Post-intervention variables

The post-intervention variables should describe the interventions or care process related to advanced airway management. These variables covers the success or fault indicators related to the procedure, the intervention description, and patient variables that can be influenced by the care process.

#### Specific data issues

Many EMS systems have trouble obtaining in-hospital data to complete follow-up or quality assurance of pre-hospital treatment, e.g., mortality data, on patients treated in the pre-hospital phase. This is often due to medico-legal or data security issues, and the patient is often "lost to follow up" as soon as the EMS personnel hand over responsibility for the patient to the hospital. Furthermore, most EMS systems feed into several different hospital systems, and follow-up is therefore logistically difficult. The expert group therefore chose to focus on variables that can be collected directly from the EMS patient contact without reliance on in-hospital data. However, the expert panel recommended that EMS systems establish methods to track the course of the patient after pre-hospital treatment.

Many system variables are fixed for a particular EMS system and do not change between patients; they can be regarded as fixed within the system. The expert panels therefore suggested that these key variables be reported at regular intervals or when they are changed, but not for each patient. These variables are not included in the core system variables but are described separately.

### The nominal group technique

The modified NGT process consisted of four steps. In the first round, the experts were supplied with the necessary background data:

- Unpublished literature review of pre-hospital airway management and outcome by one of the authors (SS)

- Recent guidelines from SSAI on pre-hospital airway management [12]

Further, they were asked to return proposals for a maximum of 15 core data variables and, in addition, optional data variables regarded as important. This first proposal was summarised and structured by the coordinators (SS, HML, DL), and the collated results were redistributed in the second round for additional comments and re-prioritisation. In the third round, a consensus meeting was held during which the expert panel first discussed and agreed on the inclusion criteria and then discussed their views on the data variables in a structured manner and finally agreed. In the fourth round, the expert panels were invited to comment on the conclusion by e-mail. Finally, all experts signed a letter of consent.

## Results

The expert panel agreed that any patient receiving advanced airway management, defined as the attempted insertion of an advanced airway adjunct or administration of ventilatory assistance, should meet inclusion criteria. Further, the expert panel agreed that advanced airway management during inter-hospital transfer should be excluded. In total, the expert panel agreed on 23 core data variables (Tables 1, 2, and 3).

**Table 1:** Core system variables

Data variable number	Data variable name	Type of data	Data variable categories or values	Definition of data variable
1	Highest Level of EMS provider on scene	Ordinal	1 = EMS non-Paramedic 2 = EMS-Paramedic 3 = Nurse 4 = Physician 5 = Unknown	Highest level of EMS provider on scene, excluding any non-EMS personnel (bystanders, family, etc)
				
2	Airway devices available on scene	Nominal	1 = Bag Mask Ventilation 2 = SAD 3 = ETT 4 = Surgical airway 5 = None 6 = Unknown	Airway devices available on scene and provider on-scene who knows how to use it (select all that apply)
				
3	Drugs for airway management available on scene	Nominal	1 = Sedatives 2 = NMBA 3 = Analgesics/opioids 4 = Local/topic anaesthetic 5 = None	Drugs used for airway management, available on scene and someone competent to administer them (select all that apply)
				
4	Main type of transportation	Nominal	1 = Ground ambulance 2 = Helicopter ambulance 3 = Fixed-wing ambulance 4 = Private or public vehicle 5 = Walk-in 6 = Police 7 = Other 8 = Not transported 9 = Unknown	Main type of transportation vehicle (if multiple selected, vehicle used for the majority of the transportation phase)
				
5	Response time	Continuous	Minutes	Time from when the Emergency Medical Communication Centre operator initiates transmission of the dispatch message to the first resource/unit time of arrival on the scene of the first unit, as reported by the first unit

EMS: Emergency Medical Service

ETT: Endotracheal tube

NMBA: Neuromuscular blocking agent

SAD: Supraglottic airway device

**Table 2:** Core patient variables

Data variable number	Data variable name	Type of data	Data variable categories or values	Definition of data variable
6	Co-morbidity	Ordinal	1 = No (ASA-PS = 1) 2 = Yes (ASA-PS = 2-6) 3 = Unknown	ASA-PS definition 1 = A normal healthy patient 2 = A patient with mild systemic disease 3 = A patient with severe systemic disease 4 = A patient with severe systemic disease that is a constant threat to life 5 = A moribund patient who is not expected to survive without the operation 6 = A declared brain-dead patient whose organs are being removed for donor purposes
7	Age	Continuous	YY or MM	Years rounded down. Ages under 1 year are reported in decimals (e.g., 6 month = 0.5 year)
8	Gender	Nominal	1 = Female 2 = Male 3 = Unknown	Patient gender
9	Patient category	Nominal	1 = Blunt trauma (incl. burns and strangulation) 2 = Penetrating trauma 3 = Non trauma (incl. drowning and asphyxia) 4 = Unknown	Dominating reason for emergency treatment
10	Indication for airway intervention	Nominal	1 = Decreased level of consciousness 2 = Hypoxemia 3 = Ineffective ventilation 4 = Existing airway obstruction 5 = Impending airway obstruction 6 = Combative or uncooperative 7 = Relief of pain or distress 8 = Cardiopulmonary arrest 9 = Other, specify	Dominating indication for airway intervention
11	Respiratory rate, initial	Continuous	Number/ Not recorded	First value recorded by the EMS provider on scene
12a	Systolic blood pressure, initial	Continuous	Number/ Not recorded	First value recorded by the EMS provider on scene
13a	Heart rate, initial	Continuous	Number/ Not recorded	First value recorded by the EMS provider on scene
14	GCS, initial (m/v/e)	Ordinal	Motor 1-6 Verbal 1-5 Eyes 1-4 Not recorded	First value recorded by the EMS provider on scene See also GCS definitions
15a	SpO_2_, initial; state: with or without supplemental O_2_	Continuous and nominal	Number/Not recorded 1 = Without supplemental O_2_ 2 = With supplemental O_2_ 3 = Unknown if supplemental O_2_	First value recorded by the EMS provider on scene

ASA-PS: American Society of Anesthesiologists physical status

EMS: Emergency Medical Service

GCS: Glasgow Coma Score

**Table 3:** Core post-intervention variables

Data variable number	Data variable name	Type of data	Data variable categories or values	Definition of data variable
16	Post-intervention ventilation	Nominal	1 = Spontaneous 2 = Controlled 3 = Mixed 4 = Unknown	How is patient ventilated following airway management? If both spontaneous and controlled, choose mixed.
12b	Post-intervention systolic blood pressure (SBP)	Continuous	Number/Not recorded	First value recorded by the EMS provider after finalised airway management
15b	Post-intervention SpO_2_	Continuous	Number/Not recorded	First value recorded by the EMS provider after finalised airway management
17a	Post-intervention EtCO_2_	Continuous	Number/Not recorded	First value recorded by the EMS provider after finalised airway management
12c	Post-intervention SBP on arrival	Continuous	Number/Not recorded	First value recorded by the EMS provider after patient arrives at hospital
13b	Post intervention heart rate	Continuous	Number/Not recorded	First value recorded by the EMS provider after finalised airway management
15c	Post-intervention SpO_2 _on arrival	Continuous	Number/Not recorded	First value recorded by the EMS provider after patient arrives at hospital
17b	Post-intervention EtCO_2 _on arrival	Continuous	Number/Not recorded	First value recorded by the EMS provider after patient arrives at hospital
18	Survival status	Nominal	1 = Dead on-scene or on arrival 2 = Alive on hospital arrival 3 = Unknown	Patient survival status: EMS treatment and on arrival at hospital
19	Attempts at airway intervention	Nominal	1 = One attempt 2 = Multiple attempts by one provider 3 = Multiple attempts by two or more providers 4 = Unknown	Number of attempts at securing the airway with a supraglottic airway device (SAD) or tracheal intubation (TI).
20	Complications	Nominal	1 = ETT misplaced in oesophagus 2 = ETT misplaced in right mainstem bronchus 3 = Teeth trauma 4 = Vomiting and/or aspiration 5 = Hypoxia 6 = Bradycardia 7 = Hypotension 8 = Other, define 9 = None recorded	Problems and mechanical complications recognised on scene and caused by airway management. Physiologic complications (5, 6, and 7) are regarded as such if they were not present before airway intervention and were recorded during or immediately after airway management. The following definitions are used: hypoxia: SpO2 < 90% bradycardia: pulse rate <60 bpm hypotension: SBP < 90
21	Drugs used to facilitate airway procedure	Nominal	1 = Sedatives 2 = NMBA 3 = Analgesics/opioids 4 = Local/topic anaesthetic 5 = None	Drugs used to facilitate the airway intervention. Select all that apply
22	Intubation success	Nominal	1 = Success on first attempt 2 = Success after more than one attempt and one rescuer 3 = Success after more than one attempt and multiple rescuers 3 = Not successful	Successful intubation defined as tube verified in the trachea. An intubation attempt is defined as attempted laryngoscopy with the intent to intubate
23	Device used in successful airway management	Nominal	1 = Bag Mask Ventilation 2 = SAD 3 = Oral TI 4 = Nasal TI 5 = Surgical airway 6 = None 7 = Unknown	Device used to manage successful airway or device in place when patient is delivered at hospital/ED

ED: Emergency Department

EMS: Emergency Medical Service

ETT: Endotracheal Tube

NMBA: Neuro Muscular Blocking Agent

## Discussion on inclusion/exclusion criteria and core data variables

### Inclusion criteria

The template should include all cases of advanced pre-hospital airway management, but the definition of this term is poorly defined. The focus of pre-hospital airway management has traditionally been on tracheal intubation (TI), but supraglottic airway devices (SAD) are increasingly popular in pre-hospital airway management [12]. In the opinion of the expert group, any airway management beyond manual opening of the airway and the use of simple adjuncts, such as a Guedel airway, should be considered as advanced airway management. This includes the use of SAD, tracheal tubes, and surgical airway techniques. In addition, the expert panel agreed that patients in need of ventilatory support generally require advanced airway management and should therefore also be included.

### Exclusion criteria

The expert panel decided that the template should focus on patients treated during so-called primary missions, defined as missions where the patient is located outside a hospital with emergency care capabilities. In secondary missions, or inter-hospital transfers, patients are often already intubated and on ventilatory support, and airway management is rarely required. In the opinion of the expert panel, these secondary transfer cases probably require a different set of variables to properly describe them and are beyond the scope of this template.

### Fixed system variables

This group of variables are regarded as fixed within the system and do not change between patients. These variables are meant to provide a picture of the population and area covered by the EMS system, but also provide some information on how the EMS system is organised (Table 4). The variables need only be documented and reported once and revised if changes occur.

**Table 4:** Fixed system variables

Data variable number	Data variable name	Type of data	Data variable categories or values	Definition of data variable
1	Population	Continuous	Number	Population count in the primary response area of the EMS
2	Area	Continuous	Number	Area in sq km or sq miles of primary response area of the EMS
3	Rural, urban, split	Nominal	1 = Urban 2 = Rural 3 = Split	Urban area defined as: "De facto population living in areas classified as urban according to the criteria used by each area or country. Data refer to 1 July of the year indicated and are presented in thousands". Rural area defined as: "De facto population living in areas classified as rural. Data refer to 1 July of the year indicated and are presented in thousands".
4	Usual tiered response	Free text	Free text	Describe briefly
5	Time intervals collected	Free text	Free text	Describe briefly
6	Service mission types	Free text	Free text	Describe briefly; e.g., mainly trauma or mixed patient population
7	Times available	Free text	Free text	Describe briefly
8	Established airway management protocols	Free text	Free text	Describe briefly
9	Airway management techniques available	Free text	Free text	Describe briefly
10	Describe type of training in airway management	Free text	Free text	Describe briefly
11	Type of tracheal tube confirmation technique	Nominal	1 = Auscultation 2 = Colorimetry 3 = Capnometry 4 = Capnography 5 = none	
12	Type of available ventilator	Free text	Free text	Describe briefly

EMS: Emergency Medical Service

### System variables

Much of the discussion regarding pre-hospital airway management revolves around who should perform the procedures [9], and recent guidelines from Scandinavia have taken a stand in the discussion [12]. The expert panel therefore agreed that it was important to include the level of the EMS provider involved in the airway management as a core variable to document the influence of the providers level on patient outcome. The provider with the highest practical competence level is recorded rather than the provider who actually performed the procedure. In the majority of cases, this is likely the same person. When this is not the case, the panel determined it most likely that the provider with the highest competence takes responsibility for the procedure whether they actually performed it or not. Supervision seems to increase the success rate of airway management [23].

Some studies suggest that the use of devices other than the tracheal tube (TT), e.g., SADs, can improve survival [24,25]. SADs are also important rescue devices when TI fails [12,26]. These devices can only be used when they are available on scene; therefore, it was agreed that the availability of devices also must be recorded (this may be a 'fixed data point' in many systems). Documenting this would also explain why, for example, in a system that is not set up for TI, a TT was not chosen as the final airway.

The use of drugs to facilitate airway management has also been debated. There is good evidence that the success rate of TI is dependent on the use of sedatives and neuromuscular blockers [27,28]. Drugs are sometimes also necessary to facilitate insertion of SADs. Since the availability of drugs is a key factor in the success of airway management, the panel agreed that this is a core variable.

There may be a relationship between the mode of transport from the scene and survival in the case of trauma patients. Some studies report an improved survival rate in trauma patients transported by helicopter compared with those transported by ground [29,30]. Others have demonstrated that transport by lay persons increases survival [31]. To what extent the transport mode influences survival in relation to pre-hospital airway management is unclear, but it might influence airway management procedures performed during transport. Because of this, the expert panel found that the main type of transport should be included as a core variable.

Shy et al. demonstrated that survival following cardiac arrest improves when the time from patient collapse to intubation is shortened [32]. It therefore seems mandatory that this time interval be recorded in the template. However, the reported times of patient collapse are often unreliable and would produce unreliable data. The panel agreed that the closest alternative would be to record response time. Studies have shown that survival improves with shorter response times [33], and the time interval is also a core variable in the Utstein template for dispatch [21].

### Patient variables

Co-morbidity represents an independent predictor of mortality after trauma [34-36] and is also useful in critically ill patients [37]. The recent update of the Utstein template for uniform reporting of data following trauma (Utstein trauma template) [17] recommended the use of the American Society of Anesthesiologists Physical Status (ASA-PS) classification system [38]. In trauma patients, ASA-PS is shown to be a strong predictor of outcome [39]. Although this system is specifically designed for recording pre-existing co-morbidity in pre-operative patients, it is easily understood and simple to use. Using similar logic [17,39], the expert panel found this scoring system appropriate for classifying co-morbidity in those patients receiving pre-hospital airway management. However, the panel recognised that the ASA-PS is unfamiliar in most pre-hospital systems and therefore recommended that only the co-morbidity categories of "no" (= ASA-PS 1), "yes" (= ASA-PS 2-6), or "unknown" be recorded as core variables. At the same time, the panel strongly recommended that the individual ASA-PS scores be recorded at least as optional data variables as soon as the ASA-PS score becomes familiar to the system. The panel also found it necessary to emphasise that the ASA-PS classification system only be used to categorise a co-morbidity that exists before the current incident [39].

Age has been shown to be an independent predictor of survival after trauma [34,40] and is an essential variable for predicting hospital mortality in critically ill patients [41]. Following the argument of the Utstein trauma template [17], the expert panel recommended that the patients' nominal age be reported as a continuous variable that is rounded down and that age under 1 year be reported in decimals. This technique is in accordance with the Utstein trauma template [17] and simplifies data handling in electronic databases, although it requires the users to translate a 12-month interval into decimals.

To the knowledge of the expert group, no studies have shown any association between gender and airway management outcome or complications. Gender is disputed as a predictor for outcome in critically ill or injured patients; some have found associations between age, gender, and outcome in trauma populations [35,42,43]. The panel however acknowledged that gender is universally reported as part of the standard population data and agreed that it should be included as a core variable.

Most studies on outcome following pre-hospital airway management are based on trauma patient populations [1]. The intention of the current template is, however, to include all patient groups receiving airway management in the pre-hospital scene because non-trauma patients make up a large proportion of the patients receiving pre-hospital airway management in many European EMS systems. Little data are available addressing the effect of pre-hospital TI on survival in non-trauma populations [1]. The expert panel, therefore, recommended that patients must be identified as trauma or non-trauma to allow this question to be explored. To avoid the misinterpretation of certain special cases, the panel decided to include burns and strangulation in the blunt trauma group and drowning and asphyxia in the non-trauma group.

The indications for airway management and especially TI have been classified into three groups: failure of airway maintenance or protection, failure of ventilation or oxygenation, and expected clinical course (that will require early intubation) [44]. Other more specific indications are established in some EMS services [45]. Still, the literature offers little support that pre-hospital airway management improves survival [1]. The expert panel, therefore, found it critically important that the template include the indication for airway intervention in the core variables and suggested a list of nine categories. This variable can hopefully provide better insight into which conditions benefit from pre-hospital airway management.

The expert panel found it most appropriate to record actual values (continuous data) of all physiological variables chosen for the core dataset. Since the focus is not only trauma patients, it would be inappropriate to record, for example, only the revised trauma score [46] (RTS) when the recording of raw data can be easily translated into the appropriate categories for different scoring systems or prediction models. Furthermore, in the case of airway management, many physiological variables represent the indications for airway intervention and markers of success or complications following intervention [47].

The panel recommended recording initial pre-intervention values (first EMS contact with patient) for systolic blood pressure (SBP), respiratory rate (RR), GCS, heart rate (HR), and SpO2.

RR, SBP, and GCS are core elements of the RTS, which has been used for many years to predict the outcome of trauma patients. The use of these variables to predict outcome in pre-hospital, non-trauma populations has not been studied to our knowledge; therefore, it is important to include them in this template for the future exploration of their predictive power in mixed populations. In the recently published Utstein trauma template [17], all of these variables are included as core variables and are also reported as actual values. RR is a well-recognised indicator of respiratory distress and may predict the need for airway intervention and ventilatory support. Pre-hospital SBP is a good predictor of severe injury [48], and although it is not a direct indicator for the need to manage the airway, changes after airway intervention may indicate cardiovascular complications. Recording of both pre- and post-intervention SBP therefore seems warranted. The same argument is valid for recording both pre- and post-intervention HR; changes in HR, e.g., bradycardia, can signal cardiovascular complications or be associated with desaturation following airway management [4,49] and should therefore be recorded before and after the intervention.

The pre-hospital GCS is a strong predictor of outcome in patients with traumatic brain injury [50]. Many regard GCS scores below 9 as an indication for intubation [51,52], but patients with traumatic brain injury and higher scores may also require intubation [45]. The panel therefore found the recording of pre-intervention GCS to be essential.

Davis et al. [53] have shown that intubation at SpO2 values below 93% causes a higher incidence of subsequent desaturation and that severe hypoxia during TI is associated with increased mortality [54]. Others have also documented that hypoxia is one of the more common complications following pre-hospital TI [4,49] and that it is useful to document SpO_2 _during pre-hospital TI [55]. The panel recommended that the initial pre-intervention SpO_2 _be recorded and that it is also recorded whether the patient was receiving supplemental O_2 _or not.

### Post-intervention variables

Poorly controlled ventilation following TI in patients with traumatic brain injury may worsen outcome [54,56,57]. There is currently little data available documenting the same effect in mixed pre-hospital trauma or non-trauma cases. Continuous monitoring of end tidal CO_2 _reduces the risk of inadvertent hyperventilation [58] and should therefore be applied in all intubated and ventilated patients pre-hospital [12]. End tidal CO_2 _monitoring is also mandatory to confirm successful TI [12,13]. The expert panel therefore recommended that the type of post-intervention ventilation be recorded and that end tidal CO_2 _values immediately after the airway intervention and on arrival in hospital be recorded as core variables.

As discussed above, SBP and SpO_2 _should be recorded pre-intervention. Both variables may also signify post-intervention complications [4,49,54] and should therefore be recorded after airway management. The panel recommended that both variables also be recorded immediately after arrival in the hospital to avoid the potential problems associated with acquiring in-hospital data.

The expert panel recommended that pre-hospital survival be the primary outcome measure for pre-hospital airway management. Many studies on pre-hospital intervention suffer from the lack of good survival data beyond the pre-hospital phase, usually as a consequence of strict rules restricting access to confidential in-hospital patient data. A recent study by Wang et al. [59] on outcome after pre-hospital intubation errors presents a novel way to link pre-hospital data with anonymous in-hospital data, but, at the same time, it illustrates the difficulty associated with achieving good survival data. The panel recommended that only survival data available from the pre-hospital phase be recorded as core data, with the variables of dead or alive on arrival at the hospital or dead on scene. Mandatory recording of in-hospital or 30-day mortality will inevitably result in some pre-hospital systems being unable to record the dataset. In systems where survival data are available, the panels recommended that 30-day survival status be collected as an optional data point [39].

Data show that multiple TI attempts are associated with a higher rate of airway-related complications [49,60]. The same may be true for SADs, and the recent SSAI guidelines recommend a maximum of three attempts of SAD insertion [12]. The panel, therefore, recommended that the total number of attempts of airway intervention be recorded, including TI and SAD attempts. To record attempts at TI specifically, the panel decided to add the data variable "intubation success", which records if TI was successful on the first attempt or after more than one attempt and if more than one rescuer was involved. In a study including 2,833 patients that received in-hospital emergency TIs outside the operating room, Mort [49] showed a significant increase in airway-related complications with three or more TI attempts. The panel agreed that it was sufficient to distinguish between one or more than one attempt in the data variable.

Complications related to airway management are probably more common in the pre-hospital setting than the in-hospital setting due to environmental, patient, and system factors [61]. One of the most severe complications reported is oesophageal misplacement of the TT [7,11,62], often with a fatal outcome. Other complications, like right mainstem TT misplacement, desaturation, regurgitation, and cardiovascular events, have also been reported [4,7,49] and can lead to increased morbidity or mortality [54]. The expert panel recommended that complications or problems related to airway management that are recognised during the pre-hospital phase be recorded as core variables. The panel has suggested seven variable categories, allowing for further categories to be defined.

The use of sedatives and neuromuscular blockers is shown to improve the success rate of pre-hospital TI [12,27,61,63]. The recent SSAI guidelines recommend the use of sedatives and neuromuscular blockers to facilitate ETI success [12]. Using SADs in patients with intact airway reflexes may also require sedation of the patient to some degree, although there is no data supporting this in the pre-hospital arena. The expert panel recommended that the use of drugs to facilitate airway management, including for the insertion of SADs, be recorded as a core variable.

Finally, the expert panel recommended the documentation of the type of device used to successfully manage the airway, meaning the device in place when the airway is regarded as successfully managed outside the hospital or the device in place on arrival at the hospital.

## General discussion

In the present paper, we present a newly developed Utstein-style template for documenting and reporting pre-hospital airway management. The expert panel reached a consensus on 23 core data variables (Tables 1, 2, and 3) that should be documented by any EMS service providing airway management in the pre-hospital setting. In addition, a set of 19 optional data variables (Additional File 1) has been discussed by the expert panel and is included in the template.

The template includes all cases of airway management where any advanced technique beyond manual airway opening and bag mask ventilation (BMV) is attempted. Further, the template includes all patient categories treated pre-hospital, not only trauma patients. These two premises are important. The increasing use of SADs in pre-hospital airway management makes it necessary to also document their use. Currently, the use of such devices pre-hospital and the impact on patient outcome are probably even less well documented than those for TI [12]. In many EMS systems, a significant proportion of the patients treated are non-trauma. Although the focus of most studies on pre-hospital airway management have been on trauma populations, a recent study from Norway showed that as many as one third of all TIs are performed on non-trauma cases, not including TI in patients with cardiac arrest (Nakstad et al. - unpublished, in review). In Adnet et al.'s data from France, more than 90% of the patients were non-trauma, when excluding those with cardiac arrest [8]. Pre-hospital airway management in these patient groups is not well documented, except in cases of cardiac arrest, where TI is not shown to influence survival according to a recent Cochrane review [1]. This review did not include the study by Shy et al. [32] mentioned above, which showed that the time to TI does impact survival. Inclusion of the entire population receiving airway management pre-hospital will hopefully provide a better understanding of the potential benefit to different patient categories.

The 19 optional data variables (Table 3) included in the template consist of variables for which the expert panel could not agree whether they should be included in the core variable set or for which the panel found that they were of such a nature that not all EMS systems would be able to document them.

In the system group, the panel agreed that the airway management experience of the provider should ideally be documented, but they could not agree on how this should be achieved. One suggestion was to indicate how many intubations the provider has performed in total, but this was felt to be a likely cause of unreliable and biased data. There are data to support that a learning curve exists for TI [64], but no studies have been performed to document this assertion in the pre-hospital arena. The suggestion to record airway management experience to record towas, however, left as an optional variable with a recommendation to document it in the systems.

The expert panel discussed the recording and documenting of the actual time of events during pre-hospital treatment. Scene times have been shown to influence survival in some studies [65,66], but there are many factors involved, and the importance of scene time vs. advanced treatment in critically ill patients remains unclear [67]. Transport times have, however, been found to influence patient outcome [29]. The panel, therefore, agreed that time events should be recorded to allow time intervals to be calculated, but recognised that it is difficult to include these as core variables at the time because differences between systems impact how time events are defined and recorded. Time events were, therefore, included in the list of optional variables.

A high body mass index seems to be related to a higher incidence of complications in airway management [68]. The recording of weight and height were, therefore, discussed, although the panel concluded that the exact documentation of these parameters is difficult in the pre-hospital phase. The variables were, however, included as optional for systems that have reliable means of recording these parameters.

Physiological variables were included in the core dataset, but a more extensive set of physiological variables was also discussed. Inclusion of a large set of variables to be recorded in the core dataset could, however, discourage users; therefore, only a limited number of variables were chosen, as discussed above. A set of optional variables was suggested for systems possessing the means to record physiological parameters more extensively and reliably.

The composition of the core and optional data points was necessarily controversial. The dataset produced was a compromise between what should be collected and what is currently practical for pre-hospital systems to collect. The entire panel agreed, for example, that in-hospital outcome data are vital information, but also recognised that including this kind of data in the template as mandatory would considerably reduce the potential use of the template. In subsequent revisions of the template, all of this data may become accessible and recordable.

Implementing an Utstein-style template is always challenging. In the case of this template, all members of the expert panel signed a letter of intention in which they agreed to recommend and work for the implementation of the core template in their systems. The experiences from these systems will form the basis of a second meeting of all experts in two years to revise the template according to experiences obtained with the first template and to additional new research. This planned revision should, however, not prevent other systems from implementing the current template. The full template with core variable definitions will therefore be available free of charge per the request of the corresponding author.

## Conclusion

We have successfully developed an Utstein-style template for documenting and reporting pre-hospital airway management. The core dataset of this template should be included in future studies on pre-hospital airway management to produce comparable data across systems and patient populations. The template will be implemented in systems influenced by the expert panel. Following a two-year period, the experiences from these systems will form the basis of a revision.

## Competing interests

The authors declare that they have no competing interests.

## Authors' contributions

SJMS, DL, and HML designed the study and organised the consensus meeting. DL was the chairman of the consensus meeting, while SJMS and HML were co-chairmen. The expert group developed the core and optional datasets. SJMS drafted the manuscript with support from DL and HML. The expert group read the manuscript once and made comments or suggested improvements before they approved it. All authors have read and approved the final manuscript.

## Supplementary Material

Additional file 1 **Optional data variables**. All 19 optional data variables with variable categories and definitions. Click here for file
