# Supplementary material for: A consensus-based template for uniform reporting of data from pre-hospital advanced airway management
Source: Scand J Trauma Resusc Emerg Med. 2009 Nov 20;17:58. doi: 10.1186/1757-7241-17-58 (PMC2785748; doi:10.1186/1757-7241-17-58)
Supplement: Additional file 1 — Optional data variables. All 19 optional data variables with variable categories and definitions. [file 1757-7241-17-58-S1.DOC]

**Additional file 1 Optional data variables**

| **Data variable number** | **Data variable name** | **Type of data** | **Data variable categories or values** | **Definition of data variable** |
| --- | --- | --- | --- | --- |
| I | Call time | Continuous | (hh:mm) | Actual time when call is answered by dispatch |
| II | Dispatch time | Continuous | (hh:mm) | Actual time when alarm is initiated for the first unit |
| III | First unit arrived | Continuous | (hh:mm) | Actual time (as reported in service) when first unit arrives on scene |
| IV | Highest level of care arrival | Continuous | (hh:mm) | Actual time (as reported in service) when unit with highest level of care arrives on scene |
| V | Leaving scene | Continuous | (hh:mm) | Actual time when patient leaves the scene (as defined by service) or time of death if dead on scene |
| VI | Arrival in first hospital | Continuous | (hh:mm) | Actual time when patient arrives in first hospital |
| VII | Arrival in final hospital | Continuous | (hh:mm) | Actual time when patient arrives in final hospital |
| *VIII* | *Number of intubations in total the provider has performed* | *The panel recognises that the experience of the provider in terms of total number of intubations performed is important but cannot agree on how this can be implemented and quantified in the current template.* | | |
|  | PATIENT |  |  |  |
| IX | ASA-PS | Ordinal | 1 = A normal healthy patient 2 = A patient with mild systemic disease  3 = A patient with severe systemic disease  4 = A patient with severe systemic disease that is a constant threat to life  5 = A moribund patient who is not expected to survive without the operation  6 = A declared brain-dead patient whose organs are being removed for donor purposes  7 = Unknown | The pre-intervention co-morbidity existing before the incident. Derangements resulting from the incident should not be considered |
| X | Weight | Continuous | Number | Estimated weight of patient in kg or lbs |
| XI | Height | Continuous | Number | Estimated height of patient in metres or inches |
| XII | Respiratory rate, lowest pre-intervention | Continuous | Number | Lowest value recorded pre-intervention by EMS on scene |
| XIII | Blood pressure, lowest pre-intervention | Continuous | Number | Lowest value recorded pre-intervention by EMS on scene |
| XIV | Heart rate, lowest pre-intervention | Continuous | Number | Lowest value recorded pre-intervention by EMS on scene |
| XV | GCS, lowest pre-intervention (m/v/e) | Ordinal | Motor 1-6 Verbal 1-5 Eyes 1-4 Not recorded | Lowest value recorded pre-intervention by EMS on scene |
| XVIa | SpO_2_, lowest recorded pre-intervention; state: with or without supplemental O_2_ | Lowest recorded by EMS provider | Number/ Not recorded 1 = Without supplemental O_2_ 2 = With supplemental O_2_ 3 = Unknown if supplemental O_2_ | Lowest value recorded pre-intervention by EMS on scene |
| XVIb | SpO_2_, last value measured before intervention | Continuous | Number | Last value recorded before airway intervention started |
| XVIc | SpO_2_, with supplemental O_2_ | Continuous | Number | Value recorded pre-intervention with supplemental O_2_ if administered |
| XVId | SpO_2_, with non-invasive PP ventilation | Continuous | Number | Value recorded pre-intervention with non-invasive positive pressure ventilation if administered |
|  | POST INTERVENTION |  |  |  |
| XVII | Survival status | Nominal | 1 = Dead 2 = Alive 3 = Unknown | Alive or dead 30 days after incident |
| XVIII | Type of primary device attempted | Nominal | 1 = Bag mask ventilation 2 = Supraglottic airway device 3 = Endotracheal tube 4 = Surgical airway 5 = none 6 = unknown | Primary airway device used in first attempt of airway management |
| XIX | Number of rescue devices attempted | Continuous | Number | Number of different devices used in airway management rescue attempt following an unsuccessful first attempt |

ASA-PS: American Society of Anaesthesiologists physical state EMS: Emergency medical service

GCS: Glasgow coma score
